# Clinical Significance of Serum Biomarkers in Pediatric Solid Mediastinal and Abdominal Tumors

**DOI:** 10.3390/ijms13011126

**Published:** 2012-01-20

**Authors:** John A. Sandoval, Linda H. Malkas, Robert J. Hickey

**Affiliations:** 1 Department of Surgery, St. Jude Children’s Research Hospital, 262 Danny Thomas Place, MS332, Room D3040G, Memphis, TN 38105, USA; 2 City of Hope Cancer Center, Duarte, CA 91010, USA; E-Mails: lmalkas@COH.org (L.H.M.); rohickey@COH.org (R.J.H.)

**Keywords:** pediatric solid tumors, mediastinal mass, abdominal mass, serum biomarkers

## Abstract

Childhood cancer is the leading cause of death by disease among U.S. children between infancy and age 15. Despite successes in treating solid tumors such as Wilms tumor, disappointments in the outcomes of high-risk solid tumors like neuroblastoma have precipitated efforts towards the early and accurate detection of these malignancies. This review summarizes available solid tumor serum biomarkers with a special focus on mediastinal and abdominal cancers in children.

## 1. Introduction

Survival rates for children with cancer have improved over the past decades [1,2]. Recent trends show that although survival rates for children with primarily leukemias and lymphomas have improved, 5-year survival rates for pediatric solid tumors have not changed over the past 2 decades. The limited numbers of clinical trials on pediatric solid malignancies as well as difficulty in discriminating and diagnosing solid masses by using standard techniques have hindered progress in this area. Therefore, it is imperative to not only accurately determine the type of masses but also detect tumor recurrences.

Serum tumor markers offer great promise in the management of children with solid malignancies and are integral to disease diagnosis and prediction of treatment response. These cancer biomarkers are biologically measured substances that are expressed by malignant tissues, circulating tumor components, or generated by the host in response to the tumor, and constitutively serve as essential tools to aid clinicians in making diagnosis, staging, and risk assessments. The nonspecific property of some markers to be differentially expressed in other tissues limits their clinical use and hampers accurate diagnosis of disease information. Although there is a wide spectrum of pediatric solid tumors, this review discusses only current trends in the use of serum biomarkers for detection and evaluation of solid tumors in the chest and abdomen of children.

## 2. Thoracic Tumors

Mediastinal tumors are relatively common in infants and children and represent a heterogeneous group of asymptomatic or potentially life-threatening neoplastic lesions that may carry complex diagnostic and therapeutic dilemmas. In general, imaging with a chest radiograph (anterior-posterior and lateral projections) and a computed tomography (CT) scan gives anatomic guidance for primary tumor location and information on mass morphology, extent, and relation to surrounding structures. Depending on tumor location within 3 different mediastinal compartments (anterior, middle, and posterior), a differential diagnosis may be made along with information on age and clinical history. Anterior mediastinal tumors include germ cell tumors (GCTs), thymic-related, cystic hygromas, and lymphomas. Common masses in the middle mediastinum are lymphomas, bronchogenic cysts, and granulomatous disease. Posterior mediastinal masses are mainly neurogenic tumors and represent the most common tumors in the mediastinum [3].

## 3. Abdominal Tumors

Pediatric solid abdominal neoplasms encompass a wide spectrum of diseases ranging from lesions causing significant morbidity and mortality [advanced stage neuroblastoma (NB)] to conditions that can be corrected by surgery alone [favorable-stage Wilms tumor (WT)]. The physician needs to determine the nature of the mass in a timely, safe, and cost-effective manner. Likely abdominal malignancies diagnosed in children include NB, WT, hepatoblastoma (HB), lymphoma, soft tissue sarcomas, and GCTs. As for thoracic masses, diagnostic imaging plays a major role in the diagnosis and management of pediatric abdominal masses, local disease staging, identification of distant metastases, and monitoring response to therapy. However, imaging techniques cannot always accurately distinguish between benign and malignant masses and may be discrepant from histologic staging [4]. Table 1 summarizes these biomarkers. In the following sections, we describe serum markers available for pediatric solid tumors in the chest and abdomen.

## 4. Serum Biomarkers in Thoracic and Abdominal Tumors

### 4.1. Pediatric Germ Cell Tumors (GCTs)

The exact incidence of pediatric GCTs is not precisely known as approximately 50% of GCTs are benign [pure mature teratoma which do not secrete α-fetoprotein (AFP) and β-human chorionic gonadotropin (β-hCG)] and go unreported. Malignant GCTs account for 3% of all childhood cancers. GCTs may develop from variations in normal differentiation (gonadal GCTs), aberrant migration (extragonadal GCTs), or their combination. Extragonadal GCTs occur at various midline locations (retroperitoneal, genital, or cranial), but most commonly affect the mediastinum and sacrococcygeal region. Morphologically, they are divided into seminomas and nonseminomatous GCTs (NSGCTs). Most NSGCTs display mixed histologies, such as embryonal, choriocarcinomas, teratomas, and yolk sac tumors (YSTs). Although predicting the biological behavior in GCTs can be confusing owing to other factors such as patient age, histologic subtype, anatomic site, and clinical stage, serum levels of alpha-fetoprotein (AFP), beta-human chorionic gonadotropin (β-hCG), and lactate dehydrogenase (LDH) are well validated in their utility as noninvasive diagnostic indicators of GCTs [21]. These markers are sensitive and specific parameters for primary diagnosis, staging, monitoring of therapeutic response and follow-up [22]. In infants and adolescents who do not have underlying hepatic disease, a significant elevation of serum AFP or β-hCG indicates significant “secreting” components of YST or choriocarcinoma, respectively, and rules out pure mature teratoma or seminoma; though seminomas may secrete minimal hCG.

#### 4.1.1. Alpha-Fetoprotein (AFP), Human Chorionic Gonadotropin (hCG), and Lactate Dehydrogenase (LDH)

The complete characterization of AFP, β-hCG, and LDH as biologic disease markers have been addressed by numerous reports over the past several decades [23–47]. As a result of the extensive history and volume of effort directed towards the in depth analysis of these three markers, the development of guidelines for the recommendations on appropriate uses for GCTs serum markers have been well established and are some of the most long-standing predictive biomarkers assays [48,49]. Tests evaluating the pattern of expression of these serum markers provides not only the standard evaluation in a suspected GCTs but also the type of mixed NSGCT, as illustrated in Table 1, they can be associated with a broad differential diagnosis and a host of other solid malignancies. The biological importance of these established markers has been reinforced by the International Germ Cell Classification Consensus (IGCCC) which divides adults into three groups: good, intermediate, and poor prognosis groups based on the presence of primary mediastinal tumor, non-pulmonary metastases, and level of tumors markers [22]. Nonetheless in the United States, pediatric GCTs have typically been divided into risk groups according to stage; grade III and IV tumors, regardless of site, have been considered as higher risk. An analysis by the Children’s Oncology Group (COG) compared the application of the adult IGCC System to pediatric malignant NSGCT. The authors sought to determine whether the tumor marker criteria developed in adults would be prognostic among pediatric patients and whether tumor marker data may be relevant in pediatric risk stratification. Results from this study showed both systems were able to stratify pediatric patients into two distinct risk groups (although the two systems are not highly concordant), were prognostic, and effective [50].

### 4.2. Hepatoblastoma (HB)

Liver malignancies account for slightly more than 1% of all pediatric tumors, with approximately 100–150 new cases of liver tumors being diagnosed annually in the US [51]. Pediatric liver tumors include hepatoblastoma (HB), hepatocellular carcinoma (HCC), sarcomas, GCTs, and rhabdoid tumors. The pediatric embryonal tumor HB accounts for 66% of malignant hepatic neoplasms.

We have previously detailed the role of raised serum AFP levels in a significant proportion of GCTs, AFP measurements also have important diagnostic implications in pediatric liver tumors, most notably HB and HCC [52]. Elevations of serum AFP also occur in non-malignant conditions such as mesenchymal hamartoma, viral and chronic active hepatitis, cirrhosis, ulcerative colitis, tyrosinemia, and in some immune-deficiency conditions [53]. Most patients with HB have extremely high serum levels of AFP [up to 100,000 ± 1,000,000 times the normal value (<10 ng/mL)], but approximately 5%–10% of patients have unexpectedly low or even normal AFP values [52]. For HB, the measurement of serum AFP levels is a valuable diagnostic test, owing to the correlation between AFP level and disease activity. Serial determinations of AFP determine the effectiveness of therapy. The half-life of the protein in the circulation is between 4 and 9 days, and levels usually return to the normal range (<10 ng/mL) by 4 ± 6 weeks of complete resection [54,55]. The failure of serum AFP levels to return to normal after surgery is an indication of either incomplete tumor resection or metastases. The importance of considering infants’ ages while interpreting AFP levels should be underscored, as age-dependent changes in serum AFP levels occur. AFP is initially synthesized in the yolk sac and is subsequently made by hepatocytes and gastrointestinal mucosa during embryogenesis; it reaches its peak concentration at approximately 12–14 weeks of gestation; and then declines thereafter, reaching normal concentration at 8–12 months of age [56]. As a result, AFP serum levels are highly elevated in neonates, and premature infants can have very high levels of AFP, which is accepted as normal (term neonates: 41,687 *l* g/L, preterm infants: 158,125 *l* g/L) [57].

Because an elevated level of AFP may not be a reliable diagnostic tumor marker in the neoinfantile age period, it has been reported that the origin of AFP is distinguishable by the measurement of a different isoform of AFP, known as the lectin fraction (AFP-L3). Recently, the usefulness of the AFP-L3 fraction was analyzed by Kinoshita *et al.* who measured the level of AFP-L3 in the pediatric HB and GCT patients [58]. They found the level of AFP-L3 fraction accurately assessed the malignant potential of the tumors evaluated and underscored the AFP-L3 fraction to be a useful marker, especially in infants suspected of harboring a hepatic or GCT malignancy.

Lastly, HB can also be associated with the inappropriate secretion of peptide hormones and other associated proteins. Marked thrombocytosis secondary to release of tumor-derived factors with thrombopoietin-like activity, growth factors, platelet-derived microparticles, factors released from bone marrow endothelial cells, and growth factors secreted by megakaryocytes can influence this process [59–61]. Likewise, ectopic βHCG production can cause precocious puberty in boys (virilizing HB) or a *forme fruste* in females [62]. A case of hormonally active HB causing both ectopic ACTH syndrome and PTH-related peptide-induced hypercalcemia has also been reported [63]. Although these paraneoplastic syndromes are not frequently linked to HB, they underscore the importance of considering these oncofetal proteins as tumor-associated rather than tumor-specific, and the diverse presentations that can pose diagnostic and therapeutic challenges in HB.

### 4.3. Lymphoma

Lymphomas are the third most common type of primary malignancy after leukemias and brain cancer. Extranodal lymphomas can be non-Hodgkin lymphomas (NHL) or Hodgkin lymphomas, and frequently involve structures in the abdomen and pelvis, including both the solid organs (liver, spleen, kidneys, and pancreas) and the hollow organs of the gastrointestinal tract. The most common lymphomas presenting within the abdomen are NHL, primarily representing Burkitt, Burkitt-like, or large B-cell lymphoma. In general, these are heterogeneous tumors with different patterns of clinical behavior and response to chemotherapy [64]. Many prognostic factors have been described in aggressive NHL: B symptoms (fever, night sweats, weight loss), performance status, age, serum LDH level, serum β_2_ microglobulin (β2M), tumor bulk, and number of nodal and extranodal sites of disease [65,66]. The localization of the tumor, its clinical appearance, its features on ultrasound and CT scans, as well as the absence of apparent regional node involvement are indicative of a neoplasm of mesenchymal origin. An accurate diagnosis may require an experienced pathologist to differentiate soft tissue lymphoma from neoplasms such as round cell sarcoma, undifferentiated carcinomas, and various myxoid spindle cell sarcomas. The following subsections review β2M, CA125, and Nm23-H1 as serum markers for extranodal lymphomas.

#### 4.3.1. β2 Microglobulin (β2M)

β2M, a nonglycosylated protein of molecular mass 11,800 Da, is synthesized by all nucleated cells and forms a small invariable light-chain moiety of the human leukocyte antigen (HLA) (-A, -B, -C) through noncovalent linkage on cell surfaces. While playing a role in antigen presentation and regulating tumor immunity, β2M protein is present at low levels in the serum, urine, and other body fluids under normal physiological conditions and is almost exclusively catabolized within the kidney. Many studies have demonstrated that the serum or urine β2M concentration increases in various malignancies such as breast cancer, prostate cancer, lung cancer, renal cancer, gastrointestinal and nasopharyngeal cancers, multiple myeloma, and especially lymphocytic malignancies such as NHL and multiple myeloma [67–73]. In these malignancies, the β2M level serves as an independent and significant prognostic factor. More specifically, the question of clinical utility of circulating levels of β2M in children with lymphomas has been addressed by Bien and Balcerska [74]. Serum soluble interleukin (IL)-2 receptor (sIL-2Ra), β2M, LDH and erythrocyte sedimentation rate (ESR) were followed as markers of diagnosis, prognosis and monitoring of response to therapy in childhood Hodgkin’s lymphoma. The levels and rates of elevated markers reflected well for diagnostics and especially for monitoring of response to therapy. The markers were also shown to be increased in subgroups of patients with unfavorable clinical features–bulky disease (increased sIL-2Ra, ESR and LDH), presence of B symptoms (increased sIL-2Ra, ESR and β2M) and higher stages of disease (elevated sIL-2Ra and LDH). Others have advocated the routine use of serum β2M be used in association with the International Prognostic Index to define prognostic subgroups in diffuse large B-cell lymphoma patients [75].

#### 4.3.2. CA125

CA125 is an antigen expressed on a 220 kDa glycoprotein produced by cells derived from coelomic epithelium (*i.e.*, peritoneum, pleura, and pericardium), epithelium of the female genital tract, mucosal cells of the stomach and colon, and mesothelial cells of serous membranes. Elevated serum concentrations of CA125 were thought occur only in individuals with ovarian carcinoma [76], but are now known to occur in many individuals with benign and neoplastic conditions such as cervical and endometrial carcinoma, benign and malignant ascites, kidney cancer, non–small cell lung cancer, breast cancer, gastric cancer, lymphoma, pelvic inflammatory disorder as well as during pregnancy [77–85]. Serum CA125 levels are high in patients with NHL, especially those with advanced disease [86]. Peritoneal mesothelial cells stimulated by the lymphokines produced by lymphoma cells are likely responsible for the high serum levels of CA125 [87,88]. Despite the enthusiasm with CA125 as a prognostic marker for NHL, the clinical utility surrounding the marker remains uncertain. A systematic review conducted by Ojha and coworkers [89] evaluated the concordance of conclusions derived from analyses of CA-125 as an independent marker for advanced stage and mortality within the same study to determine the prognostic value of CA-125 in NHL. The results derived from analyses of CA-125 as a marker for NHL are generally concordant. However, they noted a pervasive analytic misconception in previous studies precludes inferences regarding the prognostic value of CA-125.

#### 4.3.3. Nm23-H1

Nm23-H1 was originally identified as a protein that has lower expression in metastatic cancer cells than in normal cells [90]. Nm23 proteins participate in a broad spectrum of cellular responses such as development, differentiation, proliferation, oncogenesis, and tumor metastasis. The mechanisms responsible for these pleomorphic effects are varied, as it is hypothesized that Nm23 plays a tissue-specific role, given its different regulatory mechanisms in various tumors. For example, reduced *Nm23* gene expression is significantly associated with aggressive behavior in melanoma, breast, colon, and other carcinomas [91]. In contrast, *Nm23* gene expression is high in the advanced stage of thyroid carcinomas and associated with a significant decrease in survival rates for patients with NB [92]. In hematological malignancies, *NM23* genes are over expressed in acute myelogenous leukemia (AML), acute lymphoblastic leukemia (ALL), chronic myelogenous leukemia in blastic crisis (CMLBC), and myelodysplastic syndrome (MDS). Patients with aggressive lymphomas have significantly higher serum nm23-H1 levels than healthy controls do, and elevated nm23-H1 levels are associated with poor prognosis [93]. Niitsu and coworkers found that nm23-H1 expression levels in the serum and cytoplasm were significant prognostic factors in diffuse large B-cell lymphoma (DLBCL) [94] and Hodgkin lymphomas [95]. This group has also demonstrated the clinical significance of nm23-H1 in NHL [96]. These results support that serum levels of nm23-H1 may be a valuable biomarker in the diagnosis of extranodal lymphoma.

### 4.4. Neuroblastoma (NB)

NB accounts for 8% to 10% of all childhood cancers and is the most common extracranial pediatric solid tumor. This tumor remains a clinical enigma, with rates ranging from more than 90% in patients with locoregional tumors receiving little to no cytotoxic therapy to under 30% for those 18 months or older at diagnosis with metastatic disease despite receiving aggressive multimodality therapy. Age, stage, and amplification of the *MYCN* oncogene are the most validated prognostic markers. NB is designated as a neuroendocrine-related tumor because of the secretion of various regulatory peptides. The released proteins are localized in secretory granules (core- or membrane-related) in the cytosol or the cellular membrane. Proteins commonly associated with NB include secretory granule proteins chromogranin A (CgA) and synaptophysin and the cytosolic enzyme neuron-specific enolase (NSE) [97–100]. Other NB secretory proteins include neural-specific protein gene product 9.5, neuropeptide Y (NPY), vasoactive intestinal peptide (VIP), gastrin-releasing peptide, galanin, somatostatin, pancreastatin, proenkephalin-derived peptides (Met-enkephalin), peptide histidine isoleucine, pituitary adenylate cyclase–activating peptide, atrial natriuretic factor, and midkine [101–113]. The spectrum of released NB-related proteins is diverse and underscores the biological complexity involved in the regulation and modulation of this childhood tumor. Because elevated levels of serum catecholamines, CgA and NSE are important markers for NB diagnosis and prediction, we review these markers in the following subsections.

#### 4.4.1. Catecholamines

Pheochromocytoma (PCC) and NB are distinct chromaffin cell-derived tumors whose biochemical diagnoses depend on the secretion of one or several catecholamines. The plasma catecholamines dopamine (DA) and norepinephrine and their metabolites in urine, such as homovanillic acid (HVA) and vanillylmandelic acid (VMA), are diagnostic in both PCC and NB and have been used in clinical diagnosis and for early detection in screening programs in NB [114–118]. NB tumors may be differentiated on the basis of the biochemical markers released into the urine, as urinary catecholamine pattern may play different prognostic and diagnostic roles. For example, many biological parameters have been proposed to identify high-risk and low-risk NB, including a low VMA/HVA ratio, which is associated with unfavorable prognosis. Strenger *et al.* [119] evaluated the diagnostic and prognostic role of urinary catecholamines in relation to stage, age at diagnosis, and biological characteristics in 114 NB patients. His group demonstrated that low values of urinary VMA and of VMA/HVA ratios, or high values of DA/VMA and of DA/HVA ratios, may be predictive of poor prognosis in aggressive NB, being associated with MYCN amplification, 1p deletion, and other unfavorable characteristics.

#### 4.4.2. Chromogranin A (CgA) and Neuron-Specific Enolase (NSE)

Both CgA and NSE are both well established intracellular neuronal markers used to monitor NB disease activity and treatment response. Human CgA, a 48-kDa protein encompassing 439 amino acids, is an acidic glycoprotein belonging to a family of regulated secretory proteins stored in the dense core granules of the adrenal medulla and of many other neuroendocrine cells and neurons. NSE is the neuron-specific isomer of the glycolytic enzyme 2-phospho-d-glycerate hydrolyase or enolase. However, as the clinical value of serum CgA and NSE are well established for neuroendocrine tumors (NETs) [120,121], the elevation of these markers can also be observed in a number of other tumor types [122–124] and benign diseases [125–129] and therefore, do not appear as neuron-specific as considered previously.

As noted, a number of serum markers have been associated with NB in an effort to improve the clinical outcomes by facilitating the diagnosis, prognosis, or disease monitoring. In order to identify the most useful tumor markers for clinical management, Riley and colleagues [130] conducted a systematic review, and where possible meta-analyses, of molecular and biological tumor markers for NB, and attempted to establish an evidence-based perspective on their clinical value for the screening, diagnosis, prognosis, and monitoring of patients. The genetic and biologic markers identified as potentially important prognostic tools included *MYCN*, chromosome 1p, DNA index, VMA/HVA, CD44, Trk-A, NSE, LDH, ferritin, and multidrug resistance. In particular, their analyses also underscored general problems across primary tumor marker studies, in particular poor statistical and heterogeneous reporting. They emphasized better clinical interpretation and more appropriate evidence-based reviews in the future of NB biomarkers should be sought through the cooperation of cancer research groups, using multiple laboratories and standardizing methods of analysis and reporting.

### 4.5. Wilms Tumor (WT or Nephroblastoma)

Nephroblastoma is the most common primary malignant renal neoplasm in childhood, representing 6%–8% of childhood malignancies. During the last 40 years, therapeutic outcomes for WT have improved owing to large-scale multicentric and multidisciplinary efforts such as those by the National Wilms’ Tumor Study Group (currently the Renal Tumor Committee of the Children's Oncology Group) from North America and the International Society of Pediatric Oncology from Europe. These studies have served as a role model for establishing similar trials for other pediatric tumors. Current therapy options such as surgery, chemotherapy, and radiotherapy have led to an overall cure rate of approximately 85% for patients with WT. However, relapse occurs in approximately 15% of patients with favorable-histology WT and 50% those with anaplastic WT. Relapse occurs most commonly within 2 years of diagnosis and the most frequent sites are lung/pleura, tumor bed, and liver. Several studies in genetics and molecular biology have improved our understanding of this malignancy, and it is clear that a host of tumor markers such as NSE, hyaluronic acid, carcinoembryonic antigen (CEA), AFP, and even paraneoplastic syndromes that are attributable to the hormonal secretion of erythropoietin and renin are produced in WT [131]. Nevertheless, limited use of these serum markers prohibits widespread clinical applications in routine workup, detection of disease recurrence, or monitoring therapy for WT. The role of growth-related polypeptides for WT is detailed in the following section.

#### 4.5.1. Growth Factors, Cell Adhesion Molecules, and Extracellular Matrix Proteins

The associations of various growth factors, cell adhesion, and extracellular matrix proteins are valuable prognostic markers in WT [132]. In addition to these recognized factors, angiogenetic regulation plays an important role in the formation of new blood vessels involved in the growth and metastatic spread of solid tumors, but the clinical significance of using serum levels of angiogenic growth factors in WT remains to be understood. This concept has been tested, and, in theory, supports that angiogenesis regulators such as basic fibroblast growth factor (bFGF), vascular endothelial growth factor (VEGF), and hepatocyte growth factor (HGF) may be of clinical value in WT [133]. However, despite the arsenal of circulating plasma proteins associated with WT, these markers have not been routinely used. One possible reason for these blood markers being undervalued is the inconsistencies in using serum or plasma and standardized methods of collecting blood samples. For instance, VEGF is an important angiogenic stimulatory peptide essential for solid tumor growth in WT. In a review of studies on the presence of VEGF in peripheral blood, Hormbrey *et al.* [134] found that there were inconsistencies among studies with regard to the sample collection method, processing, software manipulation and data interpretation, and the specimen (plasma, serum, or whole blood) that provides the best prognostic information. Although quality-control studies are required to fully define the use of various peptides associated with WT, the discrepancies associated with tumor markers in serum or plasma may contribute to the underuse of WT-secreted proteins in the clinical setting. To date, markers identified as yet have failed to provide consistent predictive information regarding the clinical outcome of WT.

### 4.6. Soft Tissue Sarcomas

Pediatric soft tissue sarcomas are relatively rare, with 850–900 children and adolescents being diagnosed each year with rhabdomyosarcoma (RMS) or non-RMS soft tissue sarcomas (NRSTS). RMS is the most common soft tissue sarcoma in children 14 years old or younger (350 cases annually), whereas NRSTS is more predominant in adolescents and young adults. Infants also get NRSTS, but their tumors make up a distinctive set of histologies, including infantile fibrosarcoma and malignant hemangiopericytoma, which is not seen in adolescents.

RMS is a heterogenous tumor with 2 main histologic subtypes: embryonal (with botryoid and spindle cell variants) and alveolar. Approximately 60% of all newly diagnosed RMS are of embryonal histology. This subtype usually arises in mucosa-lined structures of the nasopharynx, auditory canal, and genitourinary and gastrointestinal tracts. Sarcoma botryoides, the polypoid variant of the embryonal subtype, typically arises in hollow organs and has a cluster-of-grapes appearance on pathologic inspection and imaging. Alveolar histology is seen in 20% of cases of RMS, which most commonly occurs in the trunk and extremities; the remainder have undifferentiated or pleomorphic histology [135]. Intraabdominal and pelvic RMS in children usually arises from the genitourinary system and biliary tree. Twenty percent of all cases of RMS in children involve the bladder, prostate, vagina, cervix, or paratesticular tissues and less commonly the uterus, ovary, fallopian tube, and kidney.

In contrast, a wide variety of histologic tumor types is grouped under the umbrella term NRSTS. These correspond to the various normal cell types that develop from mesenchymal cells, and, as a result, arise in a myriad of primary sites. The most common site is the extremities, followed by the trunk and abdominal regions, the thorax, and the head and neck. Surgery is a major therapeutic modality for all pediatric soft tissue sarcomas, and radiation can play a role in the local therapy for these tumors. RMS is always treated with adjuvant chemotherapy, whereas chemotherapy is reserved for high-grade or unresectable NRSTS. Although the goal of cure is realistic for the majority of patients with localized tumors, the outcome for patients who present with metastatic disease remains poor.

Sarcomas have traditionally been classified according to their morphology and type of tissue they resemble. A growing number of molecular genetic changes have been described in sarcomas, resulting in accurate categorization of these soft tissue tumors. In general, these tumors can be divided into those showing specific genetic alterations (e.g., translocations, amplifications, or activating/inactivating mutations) and those displaying multiple, complex karyotypic abnormalities with no specific pattern. In striking contrast to the added level of genetic characterization that has strengthened conventional diagnostic and management approaches for some soft tissue tumors, the role of serologic markers in sarcomas remains to be characterized. Thus far, circulating clinical markers reported include NSE [136,137], polysialylated neural cell adhesion molecule (PS-NCAM) [138], creatine kinase MB [139,140], midkine [141], osteopontin [142], and CA125 [143] for some sarcomas, but no routine serum biomarker has had efficient clinical application yet. As further efforts are required to establish the clinical role of serologic tests in this malignancy, the identification of unique serum signatures for pediatric soft tissue sarcomas may provide significant value to diagnosis, accurate prognostic prediction, and evaluation of disease and treatment progression beyond the current standard of care.

## 5. Conclusions

Serum biomarkers are useful tools to differentiate solid tumors in children. There is an abundance of literature on serum biomarkers, but this review has focused on a select handful of clinically relevant markers used for the detection, surveillance, and survival prediction of common pediatric malignancies of the chest and abdomen. Although these laboratory tests are helpful in discriminating masses, it must be recognized that some markers do not always accurately indicate disease, but are in general reliable when used in combination with other diagnostic tools. They can be used to obtain information required for planning future cancer treatment.

Nevertheless, professional societies have formal practice guidelines for the use of most tumor markers based on evidence-justified practice, which culminates pertinent, trustworthy information by systematically acquiring, analyzing, and transferring research findings into clinical, management, and policy arenas [144]. For instance, a wealth of publications on GCTs serum markers supports the use of hCG, AFP, and LDH serum concentrations for clinical management. Recent reviews by the American Society of Clinical Oncology and the National Academy of Clinical Biochemistry have outlined updated practice guidelines in adults [145,146]. These studies underscore the relevant issue of comparing age-related variation in laboratory values. Although most studies on existing GCTs tumor markers in children have similar observations, there are considerable differences in tumor biology in children and the extrapolation of results from studies in adults to children is controversial. For example, 40%–55% of GCTs in children are found at extragonadal locations as against the 5%–10% of GCTs found in adults at the same location [147–149]. This difference is likely due to variations in the maturity of the germ cells that give rise to the tumors in these age groups [150]. GCTs in children likely originate from a primordial germ cell (PGC) that underwent immediate reprogramming to become a pluripotent embryonic germ cell, whereas GCTs in adolescents and young adults most likely originate from more mature PGCs, which may be unable to survive outside of the normal niches of the ovary and testis or specialized sites such as the thymus in the case of mediastinal GCTs [151]. Additionally, the absence of BRAF mutations in pediatric GCTs in comparison to adult tumors was recently reported and further supports evidence for patient age-related biological differences [152]. This translates to distinct clinical/genetic profiles for pediatric and adult GCTs, and, especially in cases of AFP wherein these serum levels are developmentally regulated, highlights the significant role of the age of patients in determining normal baseline serum levels of some tumor markers.

Although age-adapted reference intervals are a prerequisite for proper interpretation of laboratory data, other sources of laboratory testing variation include preanalytical, total random analytical error, and intraindividual normal biology. The discrimination of different molecular forms of tumor markers may also have ramifications at the clinical level. For example, some question the validity of differences in hCG concentrations, as the recognition of hCG variants [nonnicked and nicked intact hCG (hCG and hCGn), nonnicked and nicked free β-subunit (hCGβ and hCGnβ), and regular and hyperglycosylated (or large) free hCGα] can cause discordance in hCG immunoassay results. Cao and Rej identified the following factors that contribute to the incorrect reporting of hCG results by laboratories: (a) complexity of the hCG molecule and confusion of nomenclature among various forms of hCG; (b) laboratory personnel’s lack of awareness of the distinctions in the forms of hCG and failure to recognize the specificity of assays for measurement of different forms; (c) lack of clarity and uniformity in manufacturers’ reagent labeling; and (d) lack of information on the specificity of each method for the various forms of hCG in most product inserts [153].

It is vital to accurately detect the specific hCG-related molecule in patients with malignant disease. Immunochemical characterization has shown that though intact hCG is abundantly produced by the placenta and germ cell tumors, it is the serum free β-hCG subunit (independent of the common glycoprotein hormone α subunit [GPHα]) that is also a tumor marker in many non-trophoblastic tumors, which include common epithelial cancers. Therefore, it is critical to know which hCG isoforms are measured in order to avoid false-positive results, as most automated commercial laboratory tests, point-of-care tests, and over-the-counter tests are limited in what they detect, as they focus only on regular hCG.

Despite considerable potential limitations to current pediatric tumor markers, continued progress in tumor marker discovery will likely come from rigorous translational investigations that will integrate multidimensional analyses from various emerging technologies to deliver more specific predictive and prognostic markers. Undoubtedly, next-generation biomarkers will likely stem from the growing omics technologies, which will amalgamate large-scale genomics, transcriptomics, and proteomics to promote personalized cancer care. Consequently, proteomics based biomarker discovery is one of the omics platforms that has garnered much interest in many diseases, including cancer. The enthusiasm towards cancer-related proteomics applications is due to the role proteomics can provide in bridging the gap between genetic alterations underlying cancer and cellular physiology, such that a cell’s genome is relatively stable and gives information of the organism’s gene expression while the proteome is dynamic, complex and variable. Moreover, there is a growing opinion in favor of the role of targeting proteomic measurements for improving blood-based human diagnostics [154,155]. Blood has been the logical biomarker source, as blood equilibrates with tissues and generally harbors proteins that may be identified to yield a specific proteome pattern related to a distinct pathologic process occurring in the body. To date, blood-based proteomic biomarker efforts have had little success, in large part because the relatively small number of highly abundant proteins make the reliable detection of low abundant disease-related proteins challenging due to the wide dynamic range of concentrations of proteins in a blood sample. Given the substantial complexity of plasma and the vast dynamic range of protein abundance, approaches used to simplify and increase the depth of proteomic analysis include fractionation strategies, targeted protein subpopulation enrichment (phosphoproteins, glycoproteins, *etc.*), and differential quantifying protein methods such as isobaric tags for relative and absolute quantification (iTRAQ).

In addition, other novel work in biomarker discovery involves proximal fluid proteomics. Proximal fluid, the fluid derived from the extracellular milieu of tissues, contains a large repertoire of shed and secreted proteins that are likely to be present at higher concentrations than in the plasma or serum. It has been hypothesized that many, if not all, proximal fluid proteins exchange with peripheral circulation, which has triggered the use of proximal fluids as a primary sample source for protein biomarker discovery [156].

Lastly, exciting opportunities in nanoscale exploration open new strategies for serum protein analysis. For example, Pujia and colleagues recently described a tool based on biodegradable nanoporous nanoparticles (NPNPs) that allows the harvesting of low-molecular-weight fractions of crude human serum or plasma [157]. NPNPs with a diameter of 200 nm and pore size of a few nanometers have been obtained by ultrasonication of nanoporous silicon. When incubated with a solution, NPNPs harvest only molecules that are small enough to be absorbed into the nanopores. These molecules can then be recovered by centrifugation and dissolved in water to give samples for further analyses. The development and use of novel methods in serum biomarker discovery may include strategies that use nanostructured materials and other high-throughput methods such as protein arrays, multiplexed protein assays, and chip-based proteomic platforms. For instance, utilizing protein-chip based array proteomic technologies, Wang *et al.* [158] performed protein profiling of serum samples from pre-surgery and post-surgery patients with WT and healthy controls. Two peaks (11,526 and 4,756 Da) showing significant differential expression not only between pre-surgery and control sera but also between pre-surgery and post-surgery sera were identified and characterized as serum amyloid A1 (SAA1) and apolipoprotein C-III (APO C-III).

In the continued search for ideal tumor markers in children, it is essential to pursue study designs that are appropriately powered and lend themselves to independent replication. There are several criteria for any biomarker to be considered clinically relevant. Sturgeon and colleagues recently updated the National Academy of Clinical Biochemistry laboratory medicine practice guidelines and quality requirements for the use of tumor markers. The most important feature with regard to these biomarkers is the ability to affect patient management [146]. Savage and Everett have defined the other challenges that are faced in biomarker research in children [159]. In particular, an important issue with regard to proteomics is the validation of promising candidates for pediatric diseases by blood-based profiling [160]. Proteomic profiling on children has been criticized as limited sample sizes and volumes are available for analysis, which may reduce the accuracy of the validation. Also, markers in children are discovered in the context of growth and development; few studies have reported serum or plasma proteins from healthy children. It is important to obtain serum or plasma profiles to determine age-related changes in order to better comprehend whether candidate proteins are related to disease or to normal growth. Currently, multiple profiling projects on the human plasma proteome are underway [161–163], and recent study has reported a web-based database of human plasma proteins from normal individuals [164]. Such a resource specific to children needs to be established to enable proper comparisons to interpret blood samples from healthy children and cancer patients in order to drive the identification and validation of biomarker targets.

The progress toward early diagnosis, curative therapy, and favorable outcomes in childhood solid cancer patients and survivors depends not only on developing continued strategies to identify biomarkers for more precise diagnosis, targeted therapy, and possibly prevention, but also designating additional funding or redistributing existing resources for furthering the study of childhood malignancies that continue to have static or poor survival trends. For example, 5-year survival rates for pediatric and adolescent sarcomas (Ewing, osteosarcoma, and rhabdomyosarcoma) have not drastically changed over the past 2 decades as compared with those for other solid tumors such as non–central nervous system GCTs and Wilms tumor [2]. Given the paucity of data on biomarkers and the challenging clinical management of children with metastatic sarcomas, a cross-disciplinary biological approach that comprehensively assesses DNA, RNA, protein, and metabolites to identify molecular drivers and markers is warranted to conduct small, short, and more economical individualized clinical trials. We foresee molecular data on solid pediatric cancers being translated into practical diagnostic and treatment modalities in order to optimize the success rate and hasten the implementation of effective therapies into the clinic.

## Figures and Tables

**Table 1:** Serum markers in pediatric solid tumors.

Tumor Marker	Clinically Available	Primary Cancer	Additional Associated Malignancies	Benign Diseases/Conditions	Normal Values
**AFP**	Yes	HB HCC Nonseminomatous GCT	Stomach, lung colon, and pancreatic cancer	Alcohol abuse Hepatitis Cirrhosis Biliary tract obstruction Hereditary persistence	cord: 9100–190,000 ng/mL 1 day: 7900–170,000 7 days: 3500–74,000 8–14 days: 1500–59,000 15–21 days: 580–23,000 22–28 days: 320–6300 29–45 days: 30–5800 46–60 days: 16–2000 3 months (61–90 d): 6–1000 4 months (91–120 d): 3–420 5 months (121–150 d): 2–220 6 months (151–180 d): 1–130 7 months–2 years (181–720 d): 1–87 >2 years: 1–15

**HCG**	Yes	Nonseminomatous GCT Gestational trophoblastic disease	Neuroendocrine, bladder, kidney, lung, head, neck, gastrointestinal, cervix, uterus and vulva tumors, lymphoma, and leukemia	Pregnancy Fetal Down syndrome Marijuana use Hypogonadism	<5 mU/mL (male, non-pregnant female)

**LDH**	Yes	GCT NB Lymphoma Melanoma	Small-cell lung cancer Ewing sarcoma Osteogenic sarcoma	Hemolysis Renal failure Liver and muscle diseases	0–3 days: 290–775 U/L 4–9 days: 545-2000 U/L 10 days–23 months: 180–430 U/L 2–11 years: 110–295 U/L 12–17 years: 100–190 U/L >18 years: 105–210 U/L

β**2M** [5–7]	Yes	Lymphoma	Breast, prostate, lung, renal, gastrointestinal, nasopharyngeal cancers, and multiple myeloma	Inflammatory bowel disease [8] Autoimmune diseases Acute viral infections[9]	1.1–2.4 mg/L

**CA125**	Yes	Ovary Lymphoma	Cervical and endometrial cancers, malignant ascites, renal cancer, non–small cell lung, breast, and stomach cancers, primary peritoneal carcinoma	Benign breast or ovarian disease Endometriosis Pelvic inflammatory disease Chronic renal failure Hepatitis	0–35 U/mL

**Nm23-H1**	No	Hematologic	Thyroid and NB	Psoriasis[10]	0–80 ng/mL

**Catecholamines**	Yes	NB PCC	MTC [11]	Not applicable	24-hour Urine catecholamines, fractionated, & VMA * Epinephrine, 24 hr Urine 0–2 years: Not established 3–8 years: 1–7 μg/24 h 9–12 years: 8 or less μg/24 h 13–17 years: 11 or less μg/24 h Adults: 2–24 μg/24 h
					Norepinephrine, 24 h Urine 0–2 years: Not established 3–8 years: 5–41 μg/24 h 9–12 years: 5–50 μg/24 h 13–17 years: 12–88 μg/24 h Adults: 15–100 μg/24 h
					Calculated Total (N+E) 0–2 years: Not established 3–8 years: 9–51 μg/24 h 9–12 years: 9–71 μg/24 h 13–17 years: 13–90 μg/24 h Adults: 26–121 μg/24 h Dopamine, 24 hr Urine 0–2 years: Not established 3–8 years: 80–378 μg/24 h 9–12 years: 51–474 μg/24 h 13–17 years: 51–645 μg/24 h Adults: 52–480 μg/24 h
					VMA, 24 h Urine 3–8 years: 2.3 mg or less 9–12 years: 3.4 mg or less 13–17 years: 3.9 mg or less Adults: 6.0 or less

**CgA**	Yes	NB PCC Neuroendocrine tumors	Prostate, lung, breast, gastric, and colon cancers	Hepatic disease, renal failure, rheumatoid arthritis, atrophic gastritis	0–95 ng/mL

**NSE**	Yes	NB PCC MTC Gastric or lung carcinoids	Wilms tumor Rhabdomyosarcoma	Brain hypoxia after MI, stroke, subarachnoid hemorrhage, traumatic brain damage, Guillain–Barré syndrome, bacterial meningitis and encephalitis [12–18]	0–5 nmol/mL

**Renin**	Yes	Renal (Wilms, clear cell carcinoma, and mesoblastic nephroma) [19,20]	Ovarian, lung, pancreatic, adrenal, and colon cancers	Bartter syndrome, solitary renal cyst, cirrhosis, nephrotic syndrome, dehydration Hypertension	1.9 to 3.7 ng/mL/h

Note: Abbreviations: AFP, α-feto protein; β2M, β2-microglobulin; CA125, carbohydrate antigen 125; CgA, chromogranin A; HCG, β-human chorionic gonadotropin; HCC, hepatocellular carcinoma; LDH, lactate dehydrogenase; MTC, medullary thyroid cancer; MI, myocardial infarction; NB, neuroblastoma; NSE, neuron specific enolase; Nm23-H1, nucleoside diphosphate kinase A; PCC, pheochromocytoma; VMA, vanillylmandelic acid;

* Pediatric reference ranges for catecholamines. Due to stress, plasma catecholamine levels are generally unreliable in infants and small children; urinary catecholamine assays are more reliable.
